# Heme and iron limitation in a GI-tract foundation species leads to a reshuffling of the metalloproteome and a shift toward manganese usage

**DOI:** 10.3389/fchem.2025.1562189

**Published:** 2025-04-02

**Authors:** Ronivaldo Rodrigues da Silva, James Larson, Brian Bothner, Jennifer L. DuBois

**Affiliations:** Department of Chemistry and Biochemistry, Montana State University, Bozeman, MT, United States

**Keywords:** *Bacteroides* *thetaiotaomicron*, heme, hemeprotein, iron, manganese, metalloproteome, microbiome, mass spectrometry

## Abstract

The metal-binding complement of the cellular proteome (the metalloproteome) depends on metal availability in the cellular environment and drives cellular metabolism. *Bacteroides thetaiotaomicron* (*Bacteroides theta*) is a foundational species in the anaerobic gut microbiome and a heme auxotroph, though little is known about why it requires heme. We hypothesized that *B. theta* would overproduce heme-binding proteins in response to limitations in non-heme iron, and reciprocally, activate non-heme iron pathways when heme was growth limiting. Here we showed that heme and/or non-heme iron scarcity triggers a more holistic reorganization of its metallome and metalloproteome. Under non-heme iron limitation induced by an Fe(II)-specific chelator, manganese supplementation restored growth, suggesting manganese can partly compensate for non-heme iron. Metalloproteomic analyses using tandem HPLC-ICP-MS revealed significant changes in the distribution of zinc, manganese, and iron in response to varying iron or heme availability. These findings highlight the interplay between heme/non-heme iron and the metallome in bacterial growth regulation, and they underscore a role for manganese under iron scarcity.

## 1 Introduction

Cellular life likely evolved in a marine milieu where the dominant structural elements of biochemistry–carbon, hydrogen, nitrogen, oxygen, phosphorus, and sulfur–are joined by microscopic amounts of inorganic salts and transition metals (Anbar, 2008; Remick and Helmann, 2023). These inorganic species have become essential micronutrients which cells selectively sense, incorporate, traffic, and repackage from their extracellular environments to meet cellular needs. Among transition metals, iron plays a fundamental role in biology. Iron is a cofactor in electron transport, DNA synthesis, and a variety of biosynthetic pathways, making it an essential micronutrient for nearly all forms of life (Sheldon and Heinrichs, 2015; Botta et al., 2021; Gonciarz and Renslo, 2021; Pajarillo et al., 2021). Biological iron functions either in the form of *heme*, the stable complex of Fe(II) and derivatives of the organic protoporphyrin IX (PPIX) macrocycle, or in the form of non-heme iron, a broad group of chemically labile iron species bound by protein side chains (Smith, 1984). Heme and non-heme iron constitute two functionally distinct, chemically interchangeable cellular pools. Homeostasis depends on balancing heme and non-heme iron in the presence of competing metals in dynamic chemical environments.

Here, we asked how symbiotic bacterial species adjust their total metal content (their metallomes), proteomes, and the distribution of metals bound to their proteomes (metalloproteomes), in response to changes in iron and heme availability. These changes reflect the highly dynamic gastrointestinal (GI) tract environment. We have focused specifically on *Bacteroides thetaiotaomicron (B. theta)*, a widespread model GI-tract species described as a foundation taxon because of its role in stabilizing the composition of the human gut microbiome. This anaerobe, like others in its genus and phylum, has a complex relationship with heme, which it absolutely requires but cannot make biosynthetically (Nath et al., 2024; da Silva et al., 2024). *B. theta* and related *Bacteroides* sp. must assimilate intact heme from the host environment. *B. theta* can grow with heme as its only source of iron by anaerobically metabolizing some of its heme to generate PPIX and essential free Fe(II) (Meslé et al., 2023). We previously demonstrated that this reaction is catalyzed by the product of the *hmuS* gene (Nath et al., 2024). Sources of heme and non-heme iron in the GI tract ecosystem are expected to include the host’s diet, possible feeder species within the microbiome, as well as continuously recycled intestinal cells.

We hypothesized that the *B. theta* proteome and metalloproteome would respond in distinct ways to changes in the availability of heme *versus* non-heme forms of iron. Further, because it is a heme auxotroph, we expected *B. theta* to activate heme-sparing metabolic pathways specifically in response to heme limitation. We expected these pathways would depend on non-heme iron, and that the associated changes to the (metallo)proteome would be dramatic. To test these hypotheses, we first identified growth-limiting concentrations of heme and non-heme iron. Using inductively coupled plasma mass spectrometry (ICP-MS), alone and in tandem with native size exclusion chromatography (SEC), we examined how the concentration and distribution of iron, manganese, zinc, calcium, and magnesium change in response to withholding either iron source. Limiting one form of iron or the other distinctly changed the proteome and the distribution of iron within it. Unexpectedly, however, we observed that the most dramatic changes were to the metallome and the manganese metalloproteome, including selective accumulation of manganese even when metabolizable heme was abundantly available. These observations suggest that the heme and nonheme iron regulatory and catalytic circuitry is linked to manganese metabolism by key species in the GI tract.

## 2 Materials and methods

### 2.1 Reagents and strains

Important reagents and their sources included heart Infusion broth (BD Difco), yeast extract (Fisher BioReagents), menadione (Sigma-Aldrich), hemin chloride (referred to here simply as *heme*; Calbiochem), the Fe(II)-specific chelator bathophenanthrolinedisulfonic acid disodium salt hydrate (BPS) (Thermo Scientific), MnCl_2_^.^4H_2_O (Alfa Aesar), bovine serum albumin (BSA) (Thermo Scientific), ammonium acetate (Fisher Chemical), nitric acid (EMD), and 40% BioAcryl-P (Thermo Scientific). A wild-type strain of *B. thetaiotaomicron* and a mutant strain with a disrupted *hmuS* gene were used in this study. The *hmuS* gene (located on the complement strand between nucleotide positions 608,809 and 613,201) was interrupted by a single transposon insertion at position 612,897 (GenBank accession AE015928.1). The specific strains used were *B. thetaiotaomicron* V-5482 ATCC 29148 (wild type) and the *hmuS* mutant (BT0495 P295-G04) (Arjes et al., 2022).

### 2.2 Monitoring *B. thetaiotaomicron* growth

*B. theta* glycerol stocks were utilized to prepare a pre-inoculum in a chemically defined minimal medium (MM), which contained the following components: 6.6 mM potassium dihydrogen phosphate, 15.4 mM sodium chloride, 98 µM magnesium (II) chloride hexahydrate, 176.5 µM calcium (II) chloride dihydrate, 4.2 µM cobalt (II) chloride hexahydrate, 50.5 µM manganese (II) chloride tetrahydrate, 9.3 mM ammonium chloride, 1.75 mM sodium sulfate, 134 μM L-methionine, 23.8 mM sodium bicarbonate, 8.25 mM L-cysteine (free base), and 28 mM D-glucose, adjusted to pH 7.1. The culture was incubated in Balch-type, crimp-sealed tubes (10 mL MM) for 14 h at 37°C under an anaerobic atmosphere consisting of 2.5% H_2_ and 97.5% N_2_. Subsequently, the culture was centrifuged at 3,260 ×*g* for 10 min at 4°C. The resulting cell pellet was washed twice, resuspended in MM without hemin, and used as the inoculum for monitoring growth (below).

To identify growth-limiting concentrations of heme and non-heme iron, *B. theta* cultures were grown in a nutrient rich Heart Infusion-Supplemented (HIS) medium (pH 7.5) containing 25 g L^−1^ Heart Infusion broth and 2.5 g L^−1^ yeast extract, sterilized by autoclaving (121°C, 1 atm, for 30 min). This medium was supplemented with 0.5 μg L^−1^ menadione and varying concentrations of filter-sterilized heme chloride and/or 300 µM of the Fe(II)-specific chelator BPS. This concentration of BPS was previously observed to sequester all bioavailable non-heme iron. Growth experiments were initiated with an inoculum adjusted to an optical density at 600 nm (A_600nm_) of 0.03 and conducted under anaerobic conditions (2.5% H_2_/97.5% N_2_) at 37°C with shaking at 150 rpm for 28 h (da Silva et al., 2024). Ten-milliliter cultures were grown in crimp top Balch tubes for optical monitoring *via* a UV/visible spectrometer (Genesys) or in 100 mL crimp-sealed serum bottles for preparation of larger cell pellets for protein/metal analyses (below).

### 2.3 Preparation of cell lysates

For experiments examining iron-dependent growth, wild-type *B. theta* was cultivated in 70 mL of HIS medium supplemented with either 15 µM (growth limiting) or 200 µM (excess) heme chloride, in the presence or absence of 300 µM BPS. The medium pH was adjusted to 7.5. Cells (0.3 g, or approximately 90 billion cells, Meslé et al., 2023) were collected by gentle centrifugation and resuspended in 700 µL of 100 mM ammonium acetate, pH 7.0. Cell suspensions were transferred to 2 mL FastPrep Lysis B-Matrix tubes (MP Biomedicals, Irvine, CA, USA) and subjected to mechanical lysis using a FastPrep-24 5G instrument (MP Biomedicals) at a speed of 6.0 m/s for two 40-s intervals with a 300-s pause between cycles. Lysates were clarified by centrifugation at 9,600 × *g* for 15 min at 4°C. The resulting supernatants were used for quantifying total soluble metal content and analyzing the metalloproteome.

### 2.4 Quantification of total metals in the soluble cell lysate

Samples were prepared by liberating metals from the centrifuge-clarified, soluble lysates (2.5 mg mL^−1^ total proteins) using a 20% nitric acid digestion for 30 min at 100°C (Larson et al., 2023). After centrifugation, the supernatants were diluted 15x in an acidic solution composed of 2% HNO_3_/0.5% HCl.

Soluble metals were then quantified on an Agilent 7800 ICP-MS with an Agilent SPS4 autosampler. Metal concentrations were determined using standard curves generated from serial dilution of a commercially available environmental calibration standard (CPI International). An internal standard mix (Agilent) was added to the samples using a T junction immediately before the nebulizer. The ICP-MS parameters were auto-tuned using an ICP-MS tuning solution (Agilent). The experiments were conducted using four biological replicates, and the mean values along with the standard deviation (SD) of the replicates are presented. Manganese and zinc concentrations (0.1–500 ppb), as well as magnesium, calcium, and iron concentrations (10–50,000 ppb), were subsequently converted to molarity for the purpose of graphical representation.

### 2.5 Effect of manganese on *B. thetaiotaomicron* growth

The influence of manganese on *B. theta* growth was assessed by supplementing the HIS medium, containing either 15 µM or 200 µM of heme, with 300 µM MnCl_2_·4H_2_O in the presence or absence of 300 µM BPS. Growth assays were performed under anaerobic conditions using both wild-type and *hmu* transposon mutant strains (Arjes et al., 2022). Bacterial growth was monitored spectrophotometrically by measuring A_600nm_, as described above.

### 2.6 Metalloproteome separation by size exclusion chromatography (SEC) for analyses

*B. theta* cells were grown and the soluble fractions isolated as described above. The total proteome components of the soluble fractions (diluted with 100 mM ammonium acetate (pH 7.0) to 2 mg mL^−1^ total protein concentration, determined by Bradford assay, using BSA as a standard) were fractionated in their native/folded state using size exclusion chromatography (SEC). The separation was conducted at room temperature with an Agilent Bio-SEC 3 column (3 μm, 300 Å, 4.6 × 300 mm) on an Agilent Infinity II LC integrated with an Agilent 7800 ICP-MS, using an isocratic gradient of 100 mM ammonium acetate (pH 7.0), a flow rate of 0.4 mL min^−1^, and a sample injection volume of 100 µL. Samples were kept on ice until immediately before injection. The elution profiles of five metals (^56^Fe, ^55^Mn, ^24^Mg, ^40^Ca and ^66^Zn) were acquired with an integration time/mass of 1.5 s per analyte. Monitoring of signals was done in Agilent MassHunter 4.6 (version C.01.06). In addition to the metalloproteomes, the ^56^Fe elution profiles of ferritin, and hemoglobin standards were acquired.

### 2.7 Monitoring proteome separation by size exclusion chromatography (SEC)

The total proteomes of *B. theta* cells grown under different conditions were fractionated with SEC using a high-performance liquid chromatography system (Prominence-i LC-2030C 3D Plus, Shimadzu) equipped with the same SEC column used in the SEC-ICPMS experiments. Additionally, the same mobile phase, flow rate, and injection volume was used. The autosampler was maintained at 4°C, while the column oven was set to 25°C. Proteins were monitored *via* their absorbance at 280 nm (A_280nm_) and heme-bound proteins at 400 nm. Fractions collected (1 mL per tube) from the chromatography were analyzed in parallel by SDS polyacrylamide gel electrophoresis (SDS-PAGE) with 8% acrylamide in the running gel. Metal-binding ferritin (440 kDa), Domain of Unknown Function (DUF) 2,193 from *Methanococcus voltae* (236 kDa), hemoglobin (64.5 kDa), and myoglobin (17 kDa) were used as SEC molecular weight markers and to match elution profiles between SEC-ICPMS and SEC-UVVis analyses.

## 3 Results

### 3.1 Growth-promoting and -limiting concentrations of iron sources were established for *B. thetaiotaomicron* cultures in HIS medium

We previously observed that the residual non-heme iron in a chemically defined minimal medium was sufficient to support growth of *B. theta*, if heme was supplied (15 μM). Adding 300 μM BPS removed the bioavailable non-heme iron and made heme the only source of iron in the medium, resulting in a reduced bacterial growth rate. No growth was observed in media containing 300 μM BPS and no heme (Nath et al., 2024).

**FIGURE 1:**
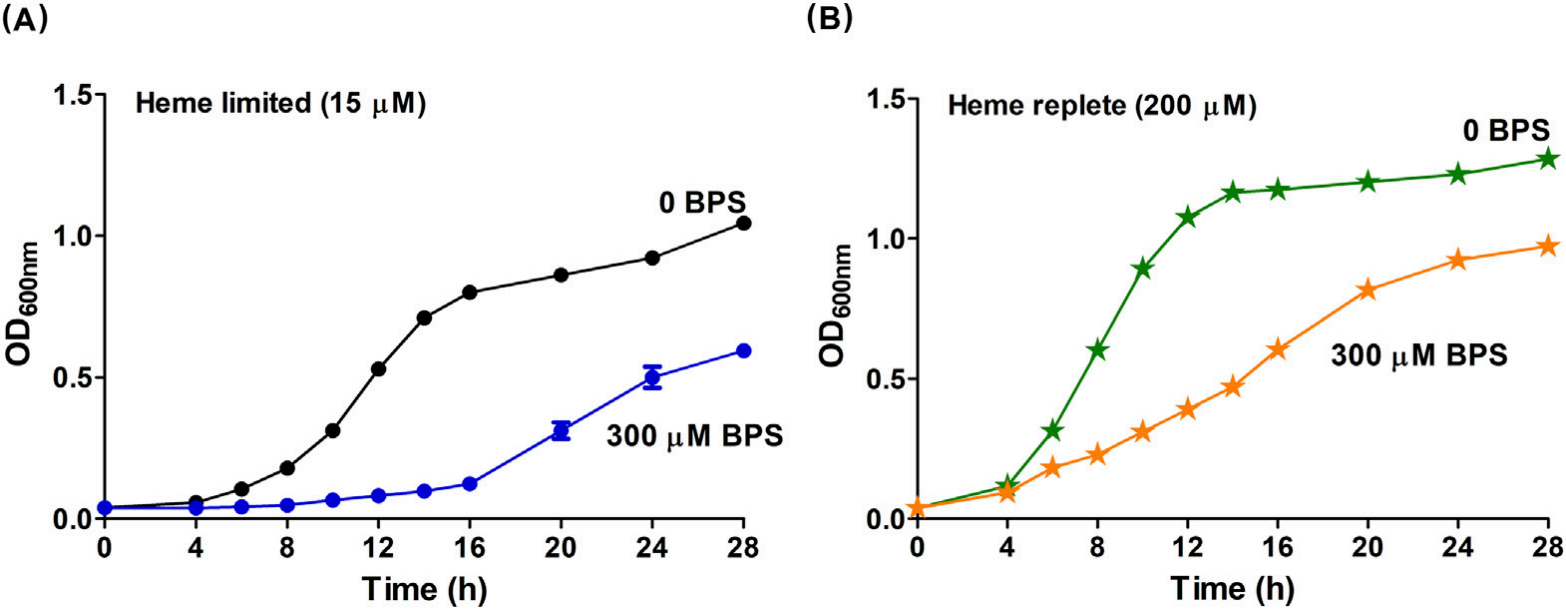
Heme stimulates the growth of *B. theta*. *B. theta* was grown in HIS media under heme limited or heme replete conditions and with different non-heme iron conditions. **(A)** Cultures in heme limited media had 15 µM heme and non-heme iron concentrations varied by the addition of BPS: Fe replete (0 µM BPS, black circles), and Fe limited (300 µM BPS, blue circles). **(B)** Cultures in heme replete media had 200 µM and non-heme Fe concentrations varied by the addition of BPS: Fe limited (0 µM BPS, green stars), and Fe replete (300 µM BPS, orange stars). The pH of HIS medium was adjusted to 7.5. Cells were grown under anaerobic conditions (2.5% H_2_/97.5% N_2_) at 37°C and 150 rpm. Experiments were carried out with four biological replicates. Averages of the replicates and standard deviations (SD) are shown.

Using similar conditions here but with a more carbon- and nutrient-rich growth medium (HIS), 300 μM BPS (a non-heme Fe-limited condition) effectively eliminated *B. theta* growth (no heme - empty square, Supplementary Figure S1A). Supplementation of this HIS + BPS medium with heme (15 μM) resulted in observable and reproducible growth (Figure 1A). Under these conditions, *B. theta* uses heme to meet cellular heme as well as non-heme iron requirements, presumptively *via* the action of the HmuS enzyme, which generates free iron and PPIX from heme (Nath et al., 2024). Consistent with that hypothesis, we observed PPIX produced by cells grown in HIS medium supplemented with 300 μM BPS and 15 μM heme (Supplementary Figure S2). We therefore designated HIS medium with 15 μM heme as Fe replete *relative to* the HIS +300 µM BPS/15 μM heme (Fe limited) condition. In each case, 15 μM heme–an amount widely used in nonselective *B. theta* growth media–was chosen to supply heme (Bacic and Smith, 2008; da Silva et al., 2024).

To identify a heme-limited growth condition, we first confirmed (in prior work) that eliminating heme from *B. theta* by serially passaging the cells into heme-free medium resulted in no culture growth (Nath et al., 2024). This result is consistent with the description of this species as a true heme auxotroph. The residual heme and non-heme iron in HIS, including any heme stored inside the cells of the inoculum, were used as a baseline to be supplemented with varying concentrations of heme (15–200 μM). We observed that *B. theta* cultures grew with rates and saturation ODs that continuously increased in proportion to the concentration of added heme, even up to the highest concentration of heme examined here (Figures 1A,B; Supplementary Figure S1B). Prior work (Meslé et al., 2023) showed that much higher concentrations of heme (≥1 mM) were needed to suppress *B. theta* growth, likely inducing a toxic response. We therefore described *B. theta* growth in Fe-sufficient HIS medium amended with 15 μM heme as limited by heme availability *(* *heme limited* *)*, relative to the HIS medium + 200 μM heme (*heme replete*) condition.

We defined a final growth condition where heme was supplied at the growth-promoting, replete concentration (200 μM) while non-heme iron was removed from the medium by chelation (300 μM BPS). Notably, experiments using this *heme replete/Fe-limited* medium yielded similar growth to cultivation in the HIS medium without added heme (Supplementary Figure S1A, 200 μM heme, and SI 1B, 0 μM heme). We expected *heme replete/Fe-limited* cultivation to maximize the use of metabolic pathways that directly require heme. The same pool of excess heme should also be available as a source of free Fe(II), but at the price of a significant metabolic expenditure, since all six genes of the *hmu* operon are likely expressed to liberate iron from heme.

### 3.2 Growth under non-heme or heme iron limitation modulated the metallome in distinct ways

**FIGURE 2:**
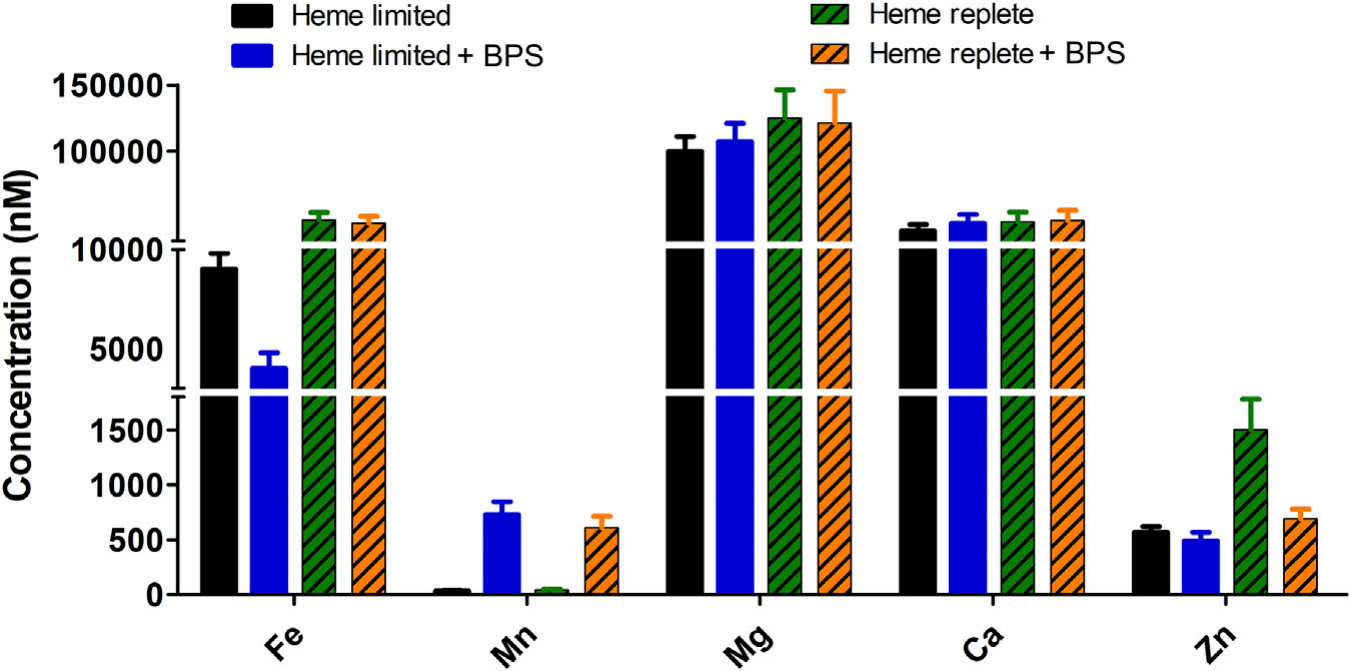
Concentration of heme and non-heme iron in growth media affects total intracellular soluble metals (^56^Fe, ^55^Mn, ^24^Mg, ^40^Ca and ^66^Zn). *B. theta* was cultivated in four different compositions of HIS medium: heme limited/non-heme Fe replete (15 µM heme + 0 µM BPS), heme limited/non-heme Fe limited (15 µM heme + 300 µM BPS), heme replete/non-heme Fe replete (200 µM heme + 0 µM BPS), heme replete/non-heme Fe limited (200 µM heme + 300 µM BPS). The media pH was adjusted to 7.5. Soluble lysate was normalized to protein concentration (2.5 mg/mL) and digested with 20% nitric acid at 100°C for 30 min. Digested lysates were analyzed with ICP-MS. Experiments were carried out with four biological replicates. Average of the replicates and standard deviation (SD) are shown.

*B. theta* cultures were grown in the different HIS-based media described above: (1) Fe replete *versus* Fe limited (each with 15 μM heme); (2) heme replete *versus* heme limited (each with sufficient non-heme iron from the HIS medium); and (3) heme replete and (non-heme) Fe limited. Equivalent masses of wet cells (0.3 g, ∼9.0 × 10^10^ cells) were lysed and their soluble components retained for each growth condition. Digestion of soluble cell lysates with nitric acid at 100°C released metals complexed with proteins, peptides, or small molecules, allowing intracellular metal content to be quantified (Figure 2). No differences between these samples were observed with respect to the quantities of the alkaline earth metals, ^24^Mg and ^40^Ca. However, all three transition metals examined exhibited varying responses to changes in the iron supply. First, ^66^Zn assimilation increased 2–3-fold specifically under the heme replete condition relative to each of the other assayed conditions. Second, roughly 4-fold greater incorporation of ^56^Fe was noted when comparing heme replete and heme limited samples. Finally, the most significant fold difference (>14) in metal incorporation was observed for manganese. Increased manganese assimilation was detected in media containing BPS (i.e., non-heme iron limited media). Comparable enhancement in ^55^Mn concentration was observed regardless of heme concentration (15 or 200 µM).

### 3.3 Under non-heme iron deprivation, manganese supplementation restored the growth of *B. thetaiotaomicron*

**FIGURE 3:**
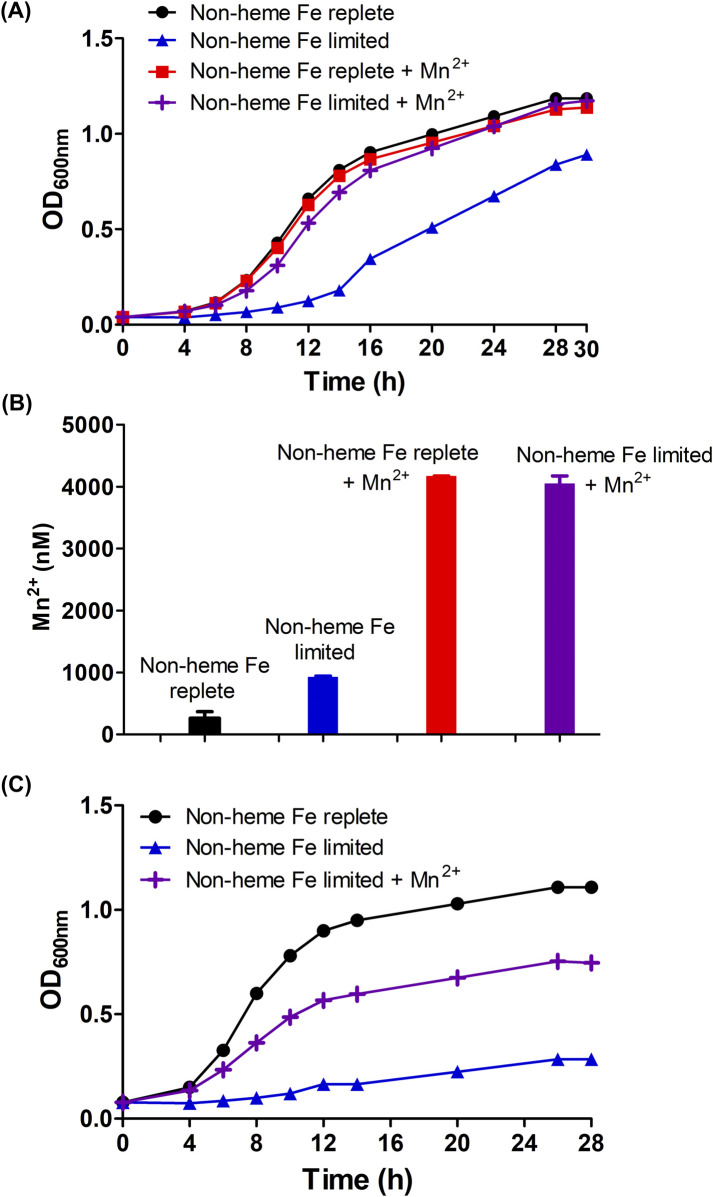
Manganese positively modulates the growth of *B. theta* (wild type and *hmuS* mutant) under non-heme iron deprivation/heme limited conditions **(A)** Wild type growth curve under test conditions **(B)** total intracellular soluble ^55^Mn from wild type cells, and **(C)** growth curve of *hmuS* mutant. *B. theta* was cultivated in four different compositions of HIS medium: non-heme Fe replete (15 µM heme + 0 µM BPS), non-heme Fe limited (15 µM heme + 300 µM BPS), non-heme Fe replete + Mn(II) (15 µM heme +0 µM BPS + 300 µM MnCl_2_^.^4H_2_O), and non-heme Fe limited + Mn(II) (15 µM heme + 300 µM BPS + 300 µM MnCl_2_^.^4H_2_O). The media pH was adjusted to 7.5. Growths were carried out under anaerobic conditions (2.5% H_2_/97.5% N_2_) at 37°C and 150 rpm. Intracellular Mn concentrations were determined by normalizing soluble lysate to protein concentration (2.5 mg/mL) and then digesting with 20% nitric acid at 100°C for 30 min. Digested lysate was analyzed with ICP-MS. Experiments were carried out with four biological replicates. Average of the replicates and standard deviation (SD) are shown.

We hypothesized that Mn(II) might be growth-promoting specifically when non-heme iron was limited by the presence of BPS. When cultured in medium containing 15 µM heme and 300 µM BPS, *B. theta* exhibited slow growth characterized by an extended lag phase of approximately 12 h and a reduced final optical density (OD_600nm_ = 0.9) (Figure 3A, blue line, triangles). Adding 300 µM MnCl_2_^.^4H_2_O (99.5% purity) to the medium restored bacterial growth to non-heme Fe-replete levels, resulting in a shorter lag phase of approximately 6 h and an increased final OD_600nm_ of 1.2 (Figure 3A, purple line, crosses). These findings indicate that manganese supplementation can restore bacterial growth in the absence of non-heme iron.

Interestingly, though *B. theta* cells do not assimilate substantial Mn(II) relative to other metals under ordinary conditions (Figure 2), and can grow well on a chemically defined minimal medium without added Mn(II), *B. theta* hyperaccumulated ^55^Mn when it was supplied at 300 μM in the growth medium. Similar uptake was observed for growth media with and without BPS (Figure 3B, red and purple bars), though a growth benefit was observed only for the non-heme iron limited condition. This suggests that: (1) Mn(II) availability to the cells was not restricted by BPS or by the presence/absence of metabolizable Fe(II), and (2) the effects of Mn(II) on cell growth were selectively exerted when Fe(II) was unavailable.

Finally, to further demonstrate a role for Mn(II) in the absence of non-heme iron, the manganese dependence of mutant *B. theta* cultures lacking a functional *hmuS* gene was measured. We previously showed that HmuS acts as a dechelatase toward heme, allowing heme to serve as a source of non-heme iron (Nath et al., 2024). When an *hmuS* (−) strain of *B. theta* was cultivated in HIS medium amended with 15 μM heme (Figure 3C, black line), the cells grew well (4 h lag time, final OD_600nm_–1). This suggests that the non-heme iron in HIS is sufficient for the mutant, which cannot harvest the iron from heme. When the same medium was depleted of non-heme iron *via* addition of 300 μM BPS, the cells failed to grow (Figure 3C, blue line, triangles). Supplementing the Fe-limited medium with 300 μM MnCl_2_⋅ 4H_2_O, however, partially restored growth (Figure 3C, purple line, crosses). This suggests that Mn(II) can rescue cells from the complete loss of non-heme iron, including iron liberated from heme.

### 3.4 Metalloproteome fractionation and metallome analyses

We hypothesized that the *B. theta* proteome, and the distribution of Fe, Mn, and Zn within it, might change in response to the different iron conditions described above. To test this hypothesis, the native/nondenatured, soluble proteomes of these cultures were size-fractionated using size exclusion HPLC (SE-HPLC). The elution profiles were monitored over time by absorbance at 280 nm (A_280nm_) to detect total proteins and by HPLC-ICP-MS to detect holoproteins containing ^56^Fe, ^55^Mn, ^24^Mg, ^40^Ca, and ^66^Zn as a function of their molecular weights.

**FIGURE 4:**
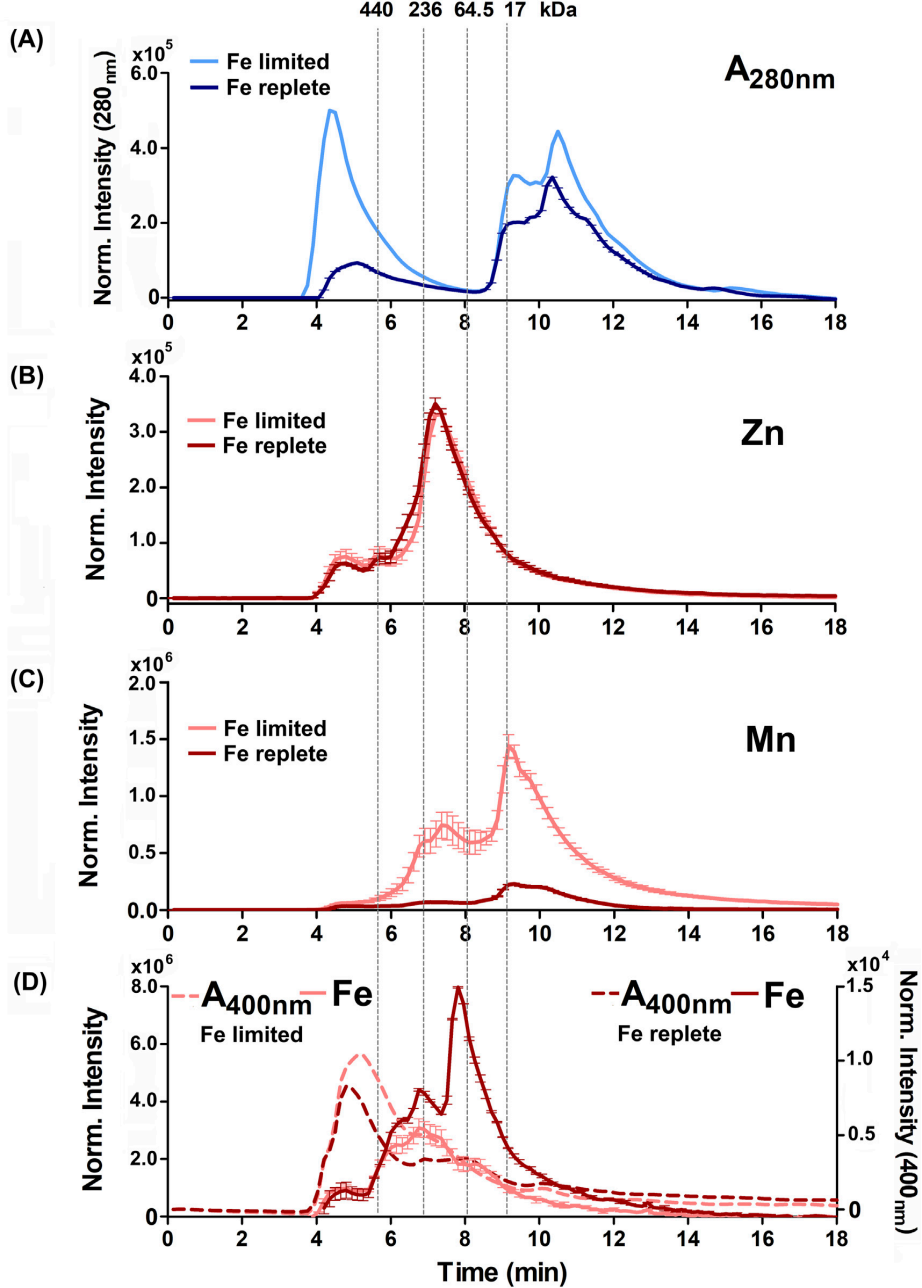
Alterations to the soluble protein, heme-binding, and metalloprotein distribution of *B. theta* under low heme concentration (15 µM heme) with replete (0 µM BPS) and deficient (300 µM BPS) non-heme iron concentrations. **(A)** SEC-UVVIS was used to measure the distribution of total proteins (A_280nm_). SEC-ICPMS was used to measure the distribution of **(B)** ^66^Zn, **(C)** ^55^Mn, and **(D)** ^56^Fe binding proteins. SEC-UVIVS was also used for the detection of heme (A_400nm_). Light shaded lines depict non-heme iron deficient (+BPS) cell lysates: protein A_280nm_ (light blue), metals (solid salmon line), and heme (A_400nm_, dashed salmon line). Dark lines depict cells grown with replete non-heme iron (no added BPS): A_280nm_ (dark blue), metals (solid red line), and heme (dashed red line). Light grey vertical lines depict the elution times of molecular weight size standards, described in the text. Experiments were carried out with two biological replicates. Averages of two replicates and standard deviation (SD) are shown.

**FIGURE 5:**
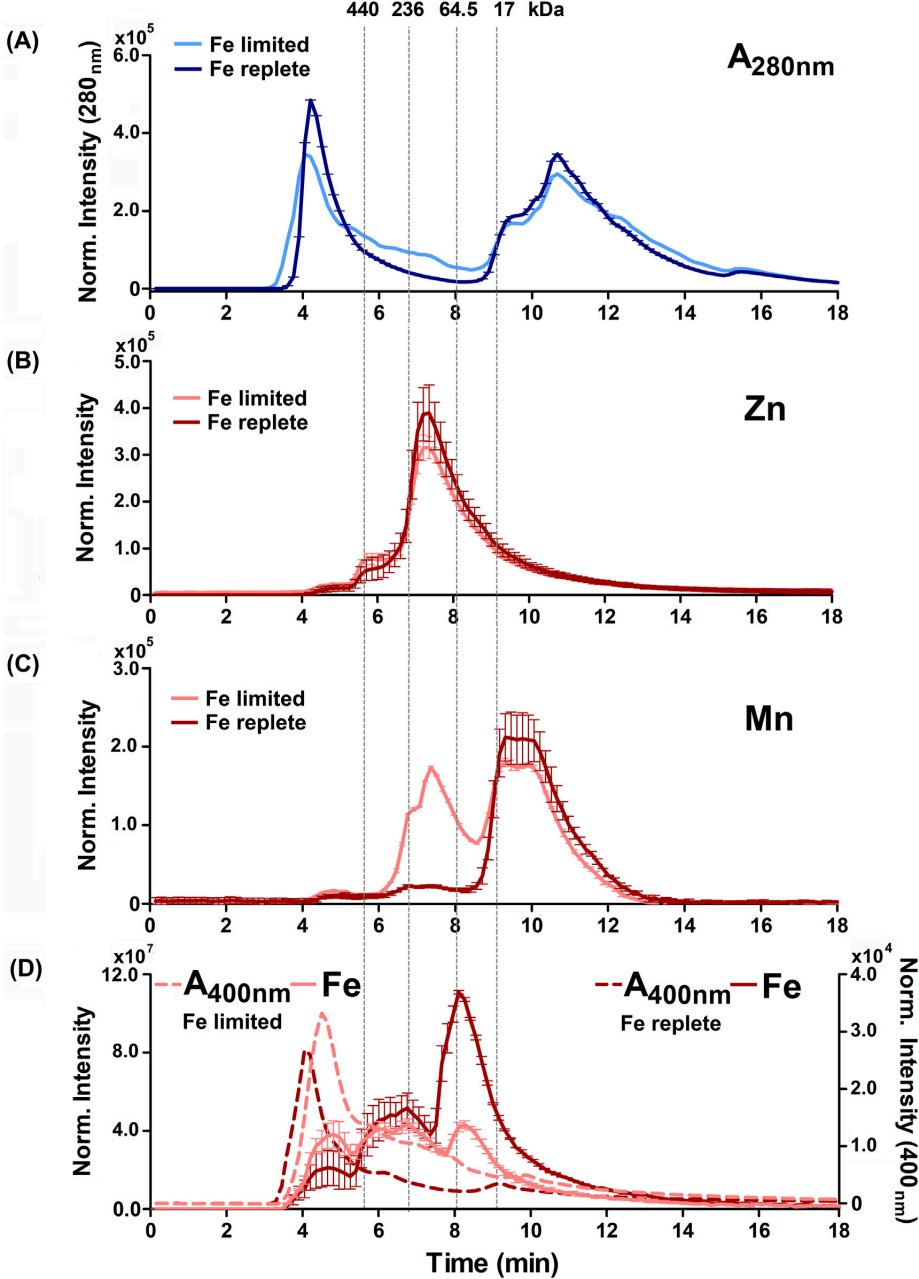
Alterations to the soluble protein, heme-binding, and metalloprotein distribution of *B. theta* under high heme concentration (200 µM heme) with replete (0 µM BPS) and deficient (300 µM BPS) non-heme iron concentrations. **(A)** SEC-UVVIS was used to measure the distribution of total proteins (A_280nm_). SEC-ICPMS was used to measure the distribution of **(B)** ^66^Zn, **(C)** ^55^Mn, and **(D)** ^56^Fe binding proteins. SEC-UVIVS was also used for the detection of heme (A_400nm_). Light shaded lines depict non-heme iron deficient (+BPS) cell lysates: protein A_280nm_ (light blue), metals (solid salmon line), and heme (A_400nm_, dashed salmon line). Dark lines depict cells grown with replete non-heme iron (no added BPS): A_280nm_ (dark blue), metals (solid red line), and heme (dashed red line). Light grey vertical lines depict the elution times of molecular weight size standards, described in the text. Experiments were carried out with two biological replicates. Averages of two replicates and standard deviation (SD) are shown.

Figures 4A, 5A show the distribution of proteins in the soluble, non-denatured proteomes of cells grown under heme limited/replete or non-heme Fe limited/replete conditions. The data are referenced to protein molecular weight size standards. These profiles illustrated subtle but reproducible changes in patterns of protein expression as a function of the availability of either iron source. SDS-PAGE analyses of the denatured proteomes reflect the subtlety of observed changes (Supplementary Figure S3). Notably, both the high-heme condition and the non-heme iron deficient condition resulted in a large, high apparent molecular weight (>440 kDa) peak in the chromatogram, eluting immediately following the column’s void volume. We suspect this fraction is due to aggregated proteins, suggesting that the cells in each case are experiencing some degree of stress due to metal limitation.

Examining the distributions of protein-associated metals (the metalloproteomes) measured with proteins in their native/folded state led to four major observations. First, we observed not only similar overall amounts (Figure 2) but also similar distributions of ^24^Mg and ^40^Ca within the size-fractionated proteins across all growth conditions examined (Supplementary Figures S4–S7). Each of these alkaline earth metals eluted with a sharp peak maximizing at approximately 9 min or 17 kDa, and a long tail that proceeded toward the lower molecular weights. Second, also consistent with the data in Figure 2, the ^66^Zn profiles were mostly insensitive to changes in the iron sources, with the exception of the heme-replete condition. In that instance, we noted increased ^66^Zn accumulation overall in the total cell cytoplasm (Figure 2) but without changes in the distribution of ^66^Zn across the metalloproteome profile (Figures 4B, 5B). Third, when non-heme iron was BPS-chelated (Fe limited conditions) against a background of either low (15 μM, Figure 4) or replete (200 μM, Figure 5) heme, we noted dramatic changes in the distributions of ^55^Mn (Figures 4C, 5C) and ^56^Fe (Figures 4D, 5D). The overall amount of ^55^Mn increased in the presence of BPS. The most pronounced increase in the proteome occurred in proteins eluting near 7.5 min (∼150 kDa). This was coupled to a change in the peak centered near 9 min (17 kDa). At the same time, the ^56^Fe-associated peak at 7–8 min diminished in the presence of the Fe(II) chelator, though the overall amount of cellular iron remained the same (Figure 2). Taken together, these results indicate increased Mn(II) uptake accompanied by redistribution of the proteome’s non-heme iron pool, when non-heme iron is restricted in the medium. Finally, we examined changes in the distribution of heme, as measured by the hemoprotein-associated Soret absorbance near 400 nm. We noted that restricting non-heme iron using BPS resulted in increased uptake of heme into the cells, with absorbance spanning a wide range of the elution profile between 4 and 11 min (Figures 4D, 5D). A major heme peak also overlapped with the protein peak eluting with a larger apparent size than the 440 kDa standard. This suggests that heme, which is well known to be limited by water insolubility, could additionally polymerize through axial water/hydroxyl ligands, and/or associate with non-polar surfaces on protein aggregates.

## 4 Discussion

Microbial metal homeostasis is perhaps best understood in the context of the host-pathogen “battle” over limited resources, where essential metals may either be withheld or deployed as weapons to stimulate production of deadly reactive oxygen species (ROS) (Chandrangsu et al., 2017). The commensal species and symbionts in the GI-tract microbiome, however, survive in an environment that is not shaped by the host immune response, but rather, by the host diet. They occupy the continuously regenerating intestinal lining. The diverse metabolic needs of the various species living communally all modulate metal availability. Prior work has shown that iron and heme deprivation affect individual bacterial physiology, while also reshaping the composition and functionality of the gut microbiota (Martin et al., 2019). We hypothesized that *B. theta*, a heme auxotroph and foundation species of the GI tract, would respond to growth-altering levels of heme restriction by reserving heme for essential pathways only, and by activating functionally redundant metabolic pathways that depend on iron in its non-heme forms. While this may be true, the effects on the cellular metallome and profiles of the metalloproteome were subtle. In contrast, readily quantifiable effects were observed when *non-heme* iron was restricted. In response to non-heme iron withholding, we measured a pronounced increase in the uptake of manganese and clear modification of both the manganese- and iron-/heme-binding proteomes.

Iron and manganese, neighbors in the first transition series, are known to interact metabolically in bacteria. Their similar ionic radii and Lewis acidity make them capable of acting interchangeably as binding partners for many biomolecules *in vitro*. Retaining function in both the Mn(II) and Fe(II) bound states is less common, except when the metal serves a Lewis acidic rather than redox role. Mn(III)/Mn(II) redox potentials are typically higher than their Fe(III)/Fe(II) counterparts (Cotruvo and Stubbe, 2013), rendering Fe(II) ideal for binding and activating O_2_ (Chandrangsu et al., 2017). As a consequence, manganese is a metal of choice when oxygen reactivity, including one-electron reduction of H_2_O_2_ to yield hydroxide and hydroxyl radical (the Fenton reaction), is either undesirable or unnecessary due to an anaerobic lifestyle (Rohaun et al., 2024). An important exception is the detoxification of superoxide (O_2_^−^) by dismutation (2O_2_^−^ → O_2_ + O_2_^2−^), which can be catalyzed by iron- or manganese-dependent superoxide dismutase enzymes (Beyer and Fridovich, 1991; Tu et al., 2012), or by cytoplasmic small Mn(II) complexes with abundant ligands such as phosphates (Lisher and Giedroc, 2013; McNaughton et al., 2010; Barnese et al., 2012).

We noted here that *B. theta* accumulates manganese when it is abundant in the growth medium, regardless of non-heme iron availability. This suggests that, unlike in *Escherichia coli*, manganese uptake is not upregulated in response to iron deprivation (for example, through induction of a manganese transporter, MntH) (Patzer and Hantke, 2001). Manganese accumulation is adaptive for pathogens, since host-induced iron limitation–by production of siderophore-sequestering lactoferrin, for example,–will not hinder the growth of a manganese-using species. This is dramatically illustrated by *Borrelia burgdorferi*, the Lyme disease pathogen, in which no iron-dependent proteins were detected in the bacterial cell lysate, and intracellular iron concentrations were found to be less than 10 atoms per cell (Posey and Gherardini, 2000). *B. burgdorferi* and species from the genera *Lactiplantibacillus* and *Lacticaseibacillus* exhibit little or no Fe(II) usage and a pronounced requirement for Mn(II), classifying them as obligately manganese-centric organisms (Lisher and Giedroc, 2013; Chandrangsu et al., 2017; Bosma et al., 2021). Proteins that typically utilize iron as a cofactor in many organisms, such as superoxide dismutase, are manganese-dependent in *B. burgdorferi* (Troxell et al., 2012).

Alternatively, manganese may become essential to otherwise “iron-centric” bacteria under specific conditions, such as oxidative stress or iron deprivation. For instance, *Escherichia coli* primarily depends on Fe(II) for normal growth but utilizes Mn(II) under conditions of oxidative stress (Anjem et al., 2009). Mechanistically, the shift to Mn(II) usage could require the expression of alternative, manganese-dependent sets of proteins (Martin and Imlay, 2011), or the use of Mn(II) as a functional substitute for Fe(II) in iron-preferring proteins (Anjem et al., 2009; Bosma et al., 2021; Capek and Vecerek, 2023).

Interestingly, unlike iron, which stays bound to and under the tight control of proteins within cells (including storage proteins like ferritins), many bacteria maintain a cytoplasmic reservoir of bioavailable manganese bound to small organic ligands (Lisher and Giedroc, 2013; McNaughton et al., 2010; Barnese et al., 2012). Even when the cells were not under non-heme iron stress, *B. theta* accumulated manganese to the same levels as stressed cells, when manganese was supplemented in the growth medium. The manganese supplementation only stimulated the growth of the iron-limited cultures. By contrast, in cells grown in regular medium, manganese levels were vanishingly low. This suggests that manganese uptake is not tightly regulated by iron deprivation, and that a reservoir of manganese may accumulate in *B. theta*. Such a reservoir, which could be comprised of the molecular weight manganese-binding species identified by HPLC-ICP-MS, could offer not only redox protection for *B. theta* living near the intestinal cell lining. It could also serve as a buffer against fluctuating concentrations of available heme and non-heme iron in the host’s diet (Anjem et al., 2009; Bosma et al., 2021; Capek and Vecerek, 2023). A manganese supply could be used to populate enzymes that are normally Fe-dependent but can function, optimally or sub-optimally, in a manganese-bound form. This interpretation seems to be a more likely explanation for enhanced growth in the presence of manganese and low iron under the strictly anaerobic conditions used in this study. Notably, uptake of dietary manganese in adult humans is low (2%–15%) (Keen and Zidenberg, 2003). This suggests that the remainder could be available for absorption by microbiome species with the capacity to use it.

Manganese may be an appropriate surrogate for iron in many reactions catalyzed by anaerobes like *B. theta*, particularly where Mn(II) is used as a Lewis acid. An obligate role for non-heme iron some proteins would still seem likely. We expect that *B. theta*, an anaerobe which encodes a wealth of predicted FeS-cluster proteins, would need to undergo shifts in expression to prioritize functionally redundant proteins with alternative cofactors (such as flavins). However, the observed growth of the *hmuS(−) B. theta* strain in the complete absence of added or heme-harvestable non-heme iron, but in the presence of manganese is remarkable. This result suggests that this species can either grow on heme/manganese alone, or that the residual non-heme iron in the inoculum used to grow the cells is sufficient to sustain the culture. This phenotype, and the identities of manganese/iron binding proteins stimulated by metal deprivation, will be the focus of future work.

From an evolutionary perspective, it is hypothesized that the adaptation of certain organisms to the interchangeable utilization of both Fe(II) and Mn(II) can be linked to the solubility and availability of these metals, which are influenced by Earth’s evolutionary history. On the early Earth, the reduced atmosphere and oceans favored the bioavailability of Fe(II). However, this condition drastically changed with the emergence of oxygenic photosynthesis by cyanobacteria (Capek and Vecerek, 2023), which led to a significant increase in the Earth’s oxygen levels approximately 2.3–2.5 billion years ago during the Great Oxidation Event (GOE) (Bekker et al., 2004; Demoulin et al., 2019; Sánchez-Baracaldo et al., 2022). As Fe(II) is more readily oxidized than Mn(II) (Capek and Vecerek, 2023), the availability of Fe(II) diminished in the presence of oxygen. It is estimated that the ratio of Fe(II) to Mn(II) decreased from 1,000:1 on primitive Earth (10^−5^ M Fe(II)/10^−8^ M Mn(II)) to 10:1 in today’s oxygen-rich oceans (10^−9^ M Fe(II)/10^−10^ M Mn(II)) (Anbar, 2008; Capek and Vecerek, 2023). Broadening the scope of metal usage to include a greater dependence on Mn(II) would have followed. In the context of the GI-tract microbiome, which lives along an O_2_ gradient spanning the fully anaerobic to the more aerobic margin of the intestinal wall, redox-controlled metal speciation and ROS production play roles that are reminiscent of life’s ancient past.

## 5 Conclusion

The dynamic interplay between microbial metal homeostasis, metal availability, and host defense mechanisms underscores the complexity of microbial survival strategies in the gastrointestinal (GI) tract. While microbial species such as *B. thetaiotaomicron* may prioritize heme utilization, non-heme iron restriction leads to significant alterations in metal uptake, notably an increase in manganese accumulation. This adaptive response highlights the complementary roles of iron, heme, and manganese in bacterial metabolism, particularly in the face of oxidative stress or nutrient limitation. Manganese, with its ability to substitute for iron in many cellular functions, offers a protective mechanism against reactive oxygen species (ROS), which are critical in the host-pathogen battle. These findings also suggest that the gut microbiome, which exists along a gradient of oxygen availability, mirrors some of the ancient biochemical processes that shaped microbial life on early Earth. Consequently, the strategic use of manganese as a substitute for iron in the microbial ecosystem may not only confer survival advantages in response to host-imposed metal restrictions but also reflects an evolutionary legacy rooted in the ancient redox conditions of Earth’s biosphere.

## Data Availability

The raw data supporting the conclusion of this article will be made available by the authors, without undue reservation.
