# Supplementary material for: Heme and iron limitation in a GI-tract foundation species leads to a reshuffling of the metalloproteome and a shift toward manganese usage
Source: Front Chem. 2025 Apr 2;13:1562189. doi: 10.3389/fchem.2025.1562189 (PMC12000045; doi:10.3389/fchem.2025.1562189)
Supplement: Supplementary file 1 [file DataSheet1.pdf]

Supplementary Material

1 Supplementary Figures and Tables

1.1 Supplementary Figures

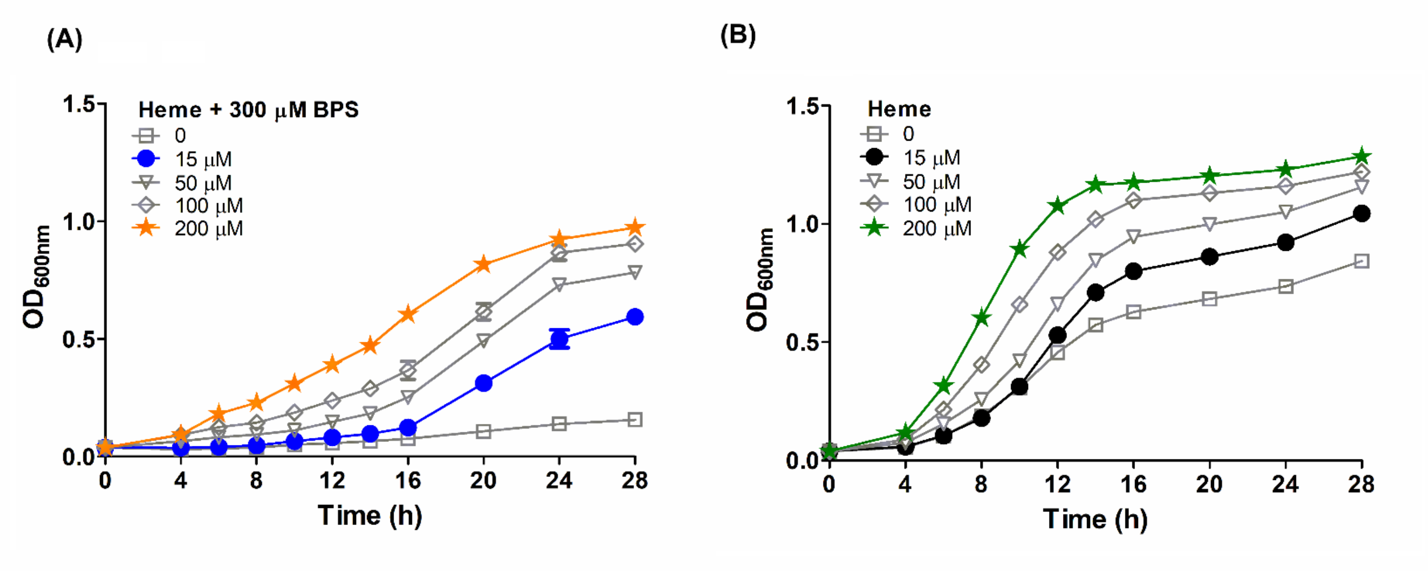

Supplementary Figure 1. Effect of different concentrations of heme on the growth of *B. theta*. *B. theta* was grown in HIS medium (**A**) with BPS, and (**B**) without BPS. The medium's pH was adjusted to 7.5. Growths were carried out under anaerobic conditions (2.5% H_2/97.5% N_2) at 37 °C and 150 rpm. Experiments were carried out with four biological replicates. Average of the replicates and standard deviation (SD) are shown.

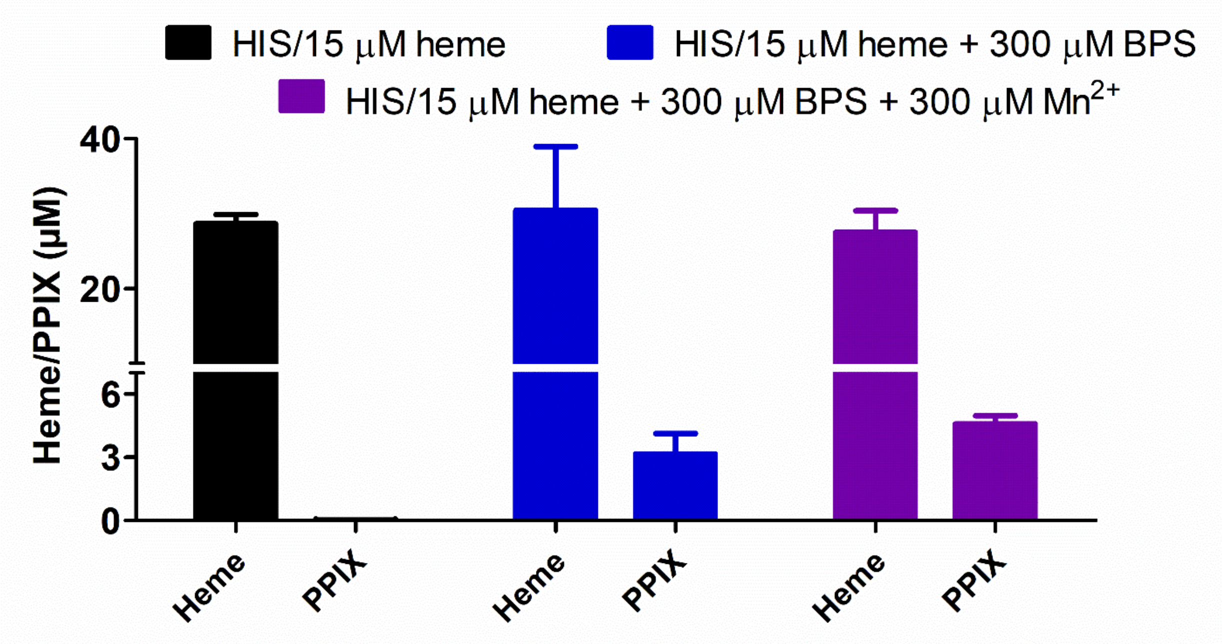

Supplementary Figure 2. Quantification of heme and protoporphyrin IX (PPIX) from *B. theta* cell lysates was performed via high-performance liquid chromatography (HPLC) at a wavelength of 400 nm, following cell lysis as described in section 2.3. *B. theta* cultures were grown under anaerobic conditions (2.5% H_2/97.5% N_2), at 37 °C, and 150 rpm in HIS medium supplemented with 15 µM heme, with or without the addition of BPS and $MnCl_2 \cdot 4H_2O$. Chromatographic separation was achieved using a Hypersil GOLD™ column (Thermo Scientific™, 4.6 mm × 250 mm, 5 µm particle size) with a linear gradient of solution A (ultrapure water + 0.1% trifluoroacetic acid, TFA) and solution B (acetonitrile + 0.1% TFA) at a flow rate of 1 mL min^{-1}, and an oven temperature of 25 °C. Experiments were conducted with two biological replicates, and the results are presented as the average values with standard deviations (SD).

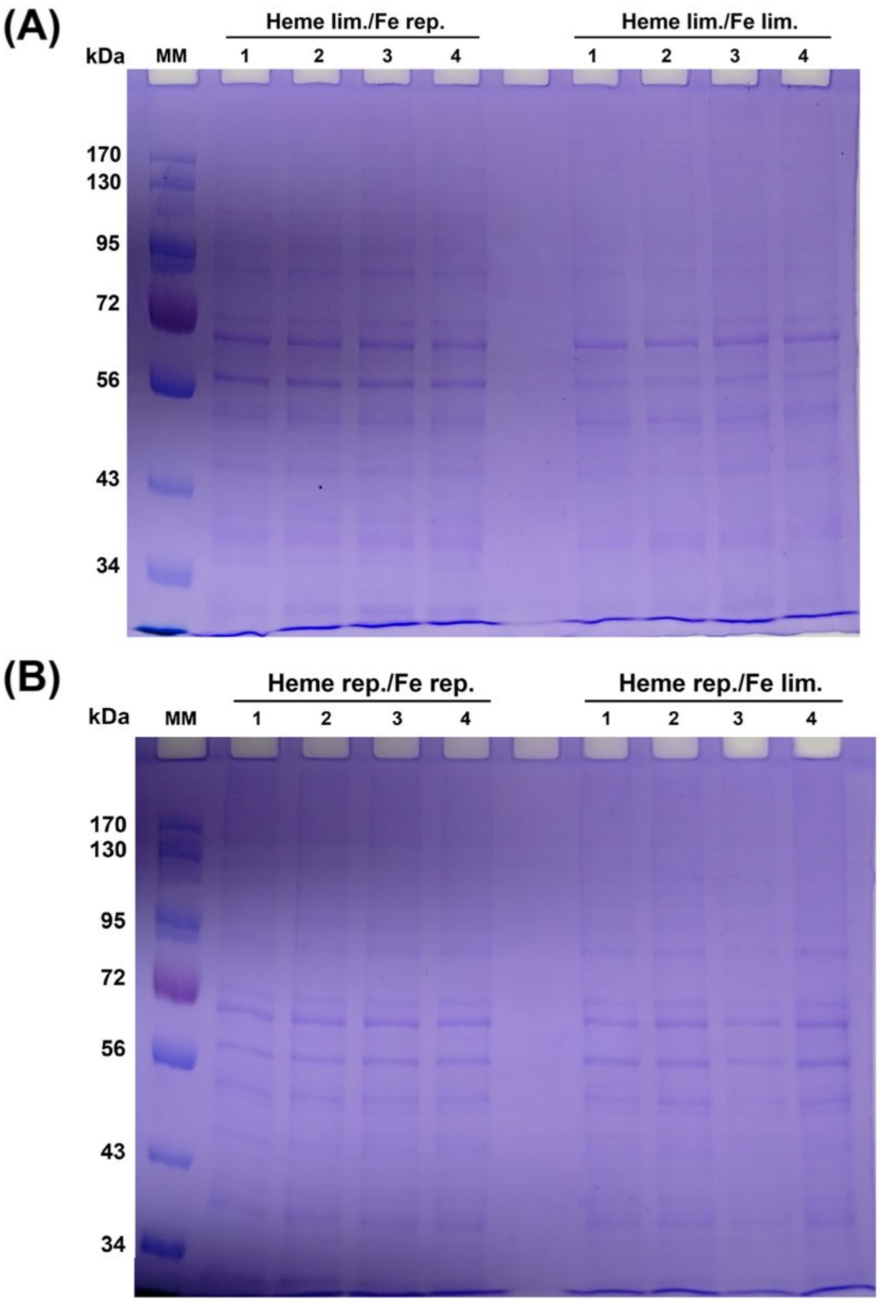

Supplementary Figure 3. Characterization of the denatured *B. theta* proteome. The soluble proteome (clarified lysate) from *B. theta* was grown in four different compositions of HIS medium; (**A**) Heme limited (15 μM) and (**B**) heme replete (200 μM) concentrations, with replete (0 μM BPS) and deficient (300 μM BPS) non-heme iron concentrations was analyzed with an 8% SDS-PAGE gel.

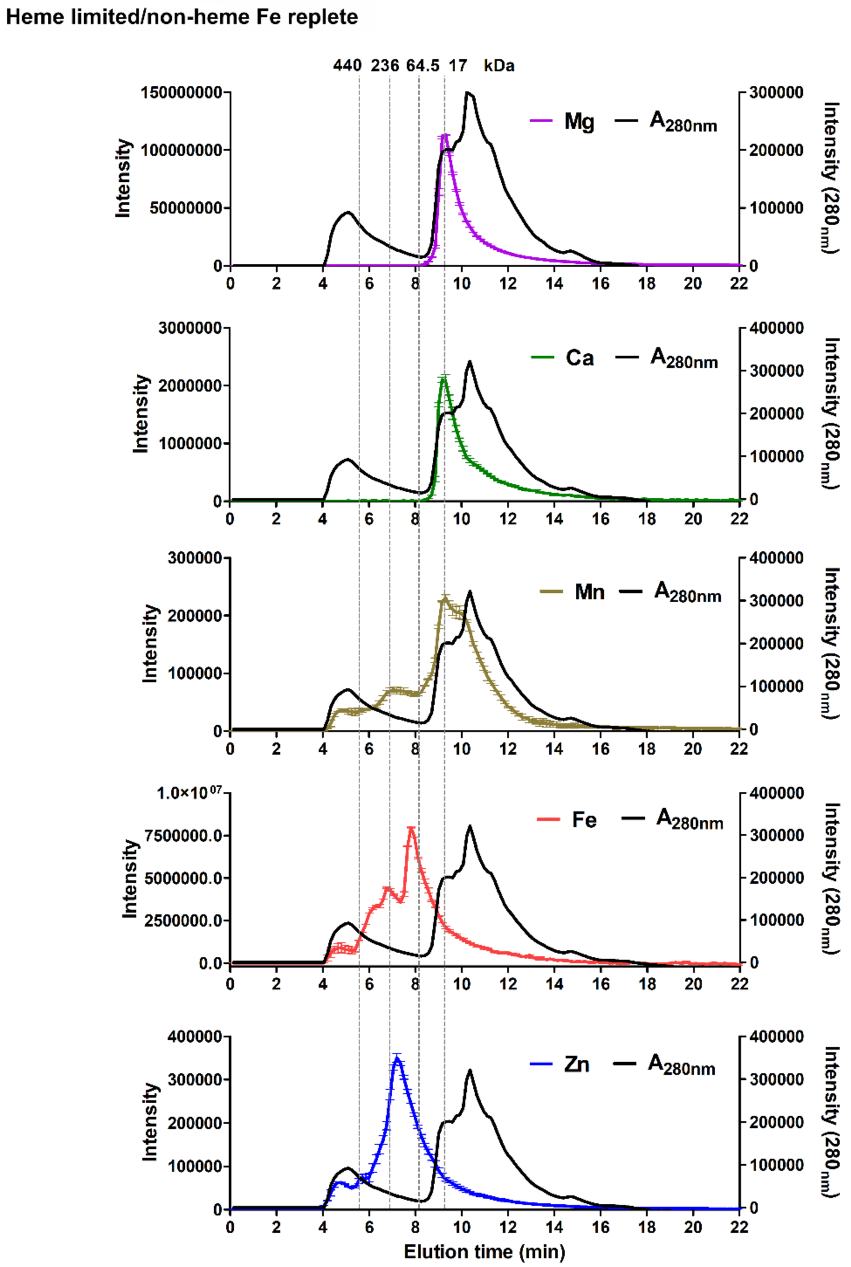

Supplementary Figure 4. Characterization of the native (metallo)proteomes of *B. theta* grown under heme limited concentration (15 µM) without BPS (***heme limited/Fe replete*** condition). *B. theta* cultures were grown under anaerobic conditions (2.5% H_2/97.5% N_2) at 37 °C, and at 150 rpm. Eluted proteins were monitored at 280 nm via SEC-UVVIS and metals (^{56}Fe, ^{55}Mn, ^{24}Mg, ^{40}Ca and ^{66}Zn) were detected via SEC-ICPMS. Experiments were carried out with two biological replicates. Average of the replicates and standard deviation (SD) are shown.

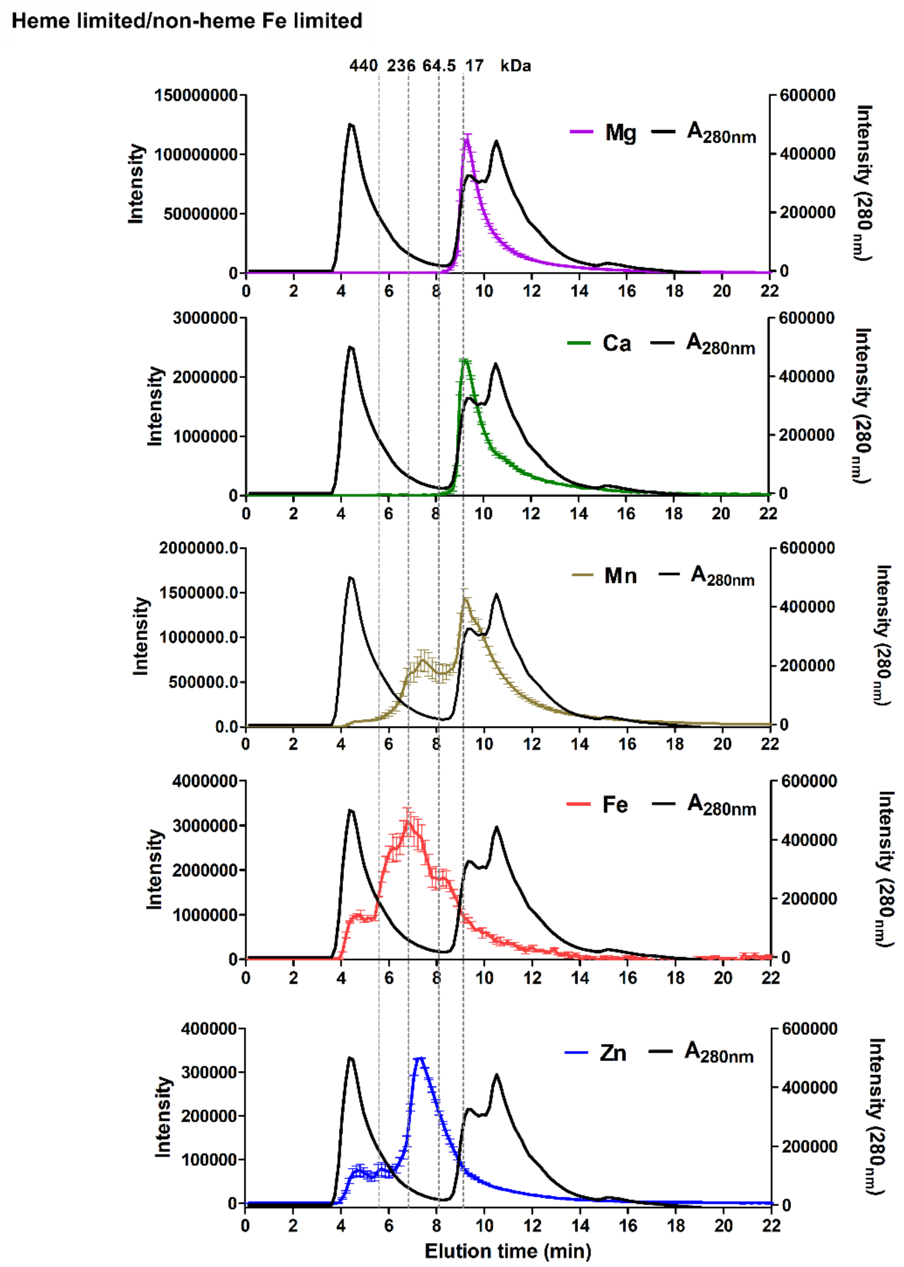

Supplementary Figure 5. Characterization of the native (metallo)proteomes of *B. theta* grown under low heme concentration (15 µM) with 300 µM BPS (***heme limited/Fe limited*** condition). *B. theta* cultures were grown under anaerobic conditions (2.5% H_2/97.5% N_2) at 37 °C, and 150 rpm. Eluted proteins were monitored at 280 nm via SEC-UVVIS and metals (^{56}Fe, ^{55}Mn, ^{24}Mg, ^{40}Ca and ^{66}Zn) were detected with SEC-ICPMS. Experiments were carried out with two biological replicates. Average of the replicates and standard deviation (SD) are shown.

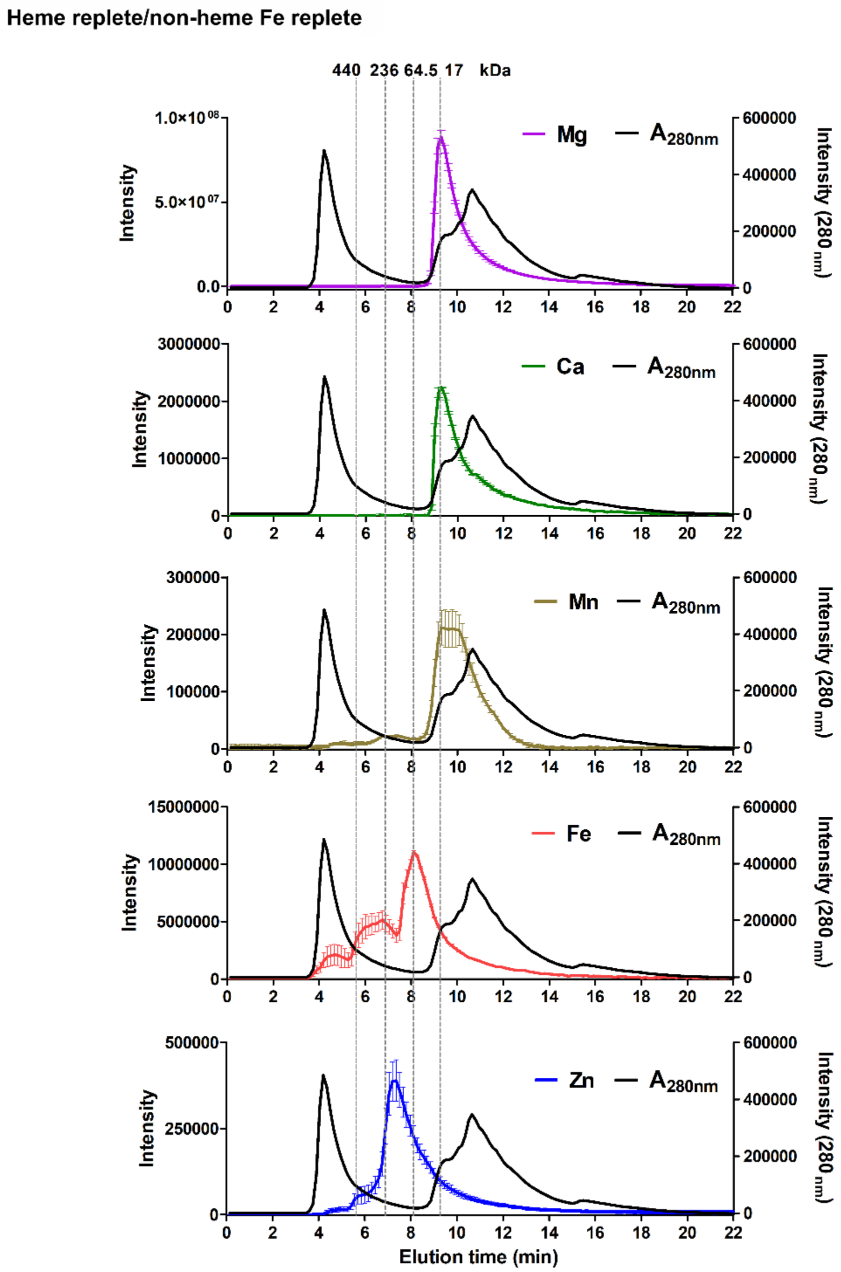

Supplementary Figure 6. Characterization of the native (metallo)proteome of *B. theta* grown under high heme concentration (200 µM) without BPS (***heme replete/Fe replete*** condition). *B. theta* cultures were grown under anaerobic conditions (2.5% H_2/97.5% N_2) at 37 °C, and 150 rpm. Eluted proteins were monitored at 280 nm via SEC-UVVIS and metals (^{56}Fe, ^{55}Mn, ^{24}Mg, ^{40}Ca and ^{66}Zn) were detected with SEC-ICPMS. Experiments were carried out with two biological replicates. Average of the replicates and standard deviation (SD) are shown.

Heme replete/non-heme Fe limited

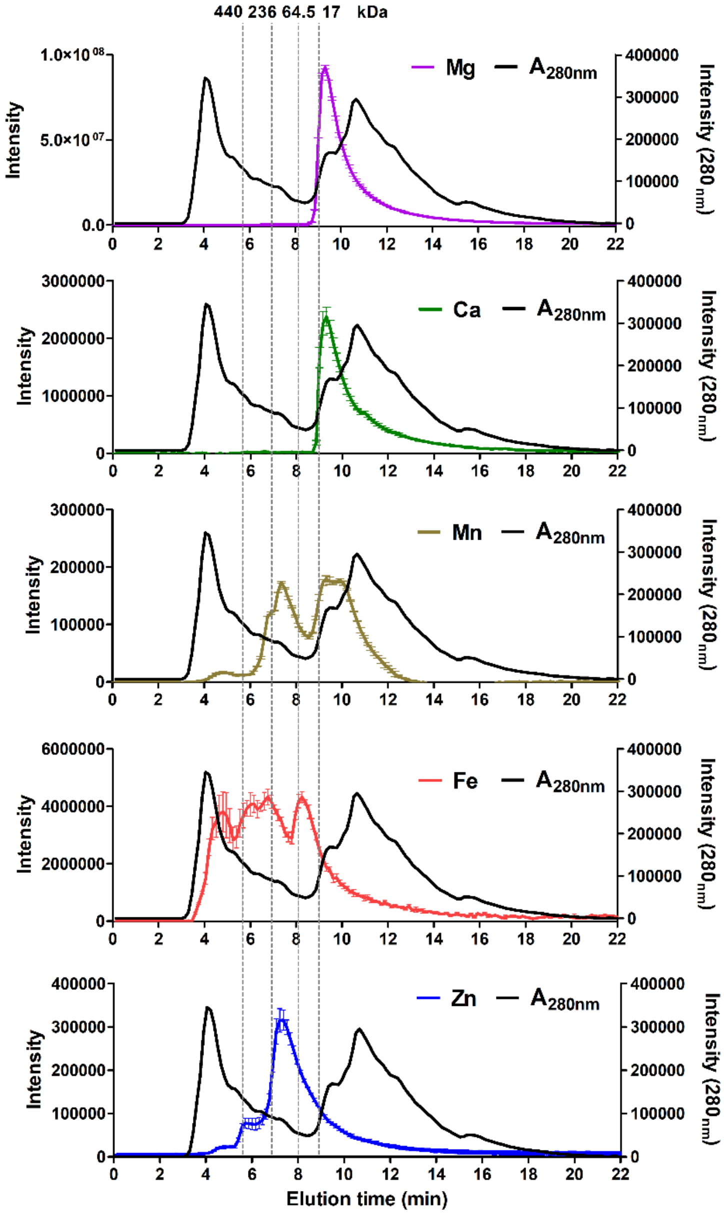

Supplementary Figure 7. Characterization of the native (metallo)proteome of *B. theta* grown under high heme concentration (200 µM) with 300 µM BPS (***heme replete/Fe limited*** condition). *B. theta* cultures were grown under anaerobic conditions (2.5% H_2/97.5% N_2) at 37 °C, and 150 rpm. Eluted proteins were monitored at 280 nm via SEC-UVVIS and metals (^{56}Fe, ^{55}Mn, ^{24}Mg, ^{40}Ca and ^{66}Zn) were detected with SEC-ICPMS. Experiments were carried out with two biological replicates. Average of the replicates and standard deviation (SD) are shown.
